# Supplementary material for: Probabilistic mapping of sub-genic intolerance reveals functional and disease-critical protein regions
Source: bioRxiv. 2026 Sep 16:2026.09.13.745535. Preprint. [Version 1] doi: 10.64898/2026.09.13.745535 (PMC13596272; doi:10.64898/2026.09.13.745535)
Supplement: Supplementary file 1 [file media-1.pdf]

Querying the OMIM Database

To obtain the “All OMIM” (n=4,270 genes) dataset, the full genemap was downloaded and filtered for genes with a phenotype that did not include the terms “resistance”, “cancer”, “somatic”, “susceptibility”, “carcinoma”, and “tumor” or symbols “{“, “[]”, and “?”, as outlined by Petrovski et al.[1]. The “Autosomal Dominant” (n=1,606) and “Autosomal Recessive” (n=2,830) genes were extracted by further filtering the full dataset for phenotypes containing the term “Autosomal dominant” and “Autosomal recessive”, respectively. The “De Novo” (n=1,305), “Dominant-Negative” (n=676), “Gain-of-Function” (n=486), and “Haploinsufficient” (n=277) gene sets were compiled by querying the terms “de novo”, “dominant-negative”, “gain-of-function”, and “haploinsufficiency”, filtering only for gene entries annotated with “*” (known sequence) and “#” (phenotype description and molecular basis known). Records were then further restricted to require “Allelic variants” and a “Gene Map Locus”. The overlap among these gene sets is summarized in Supplementary Table 7.

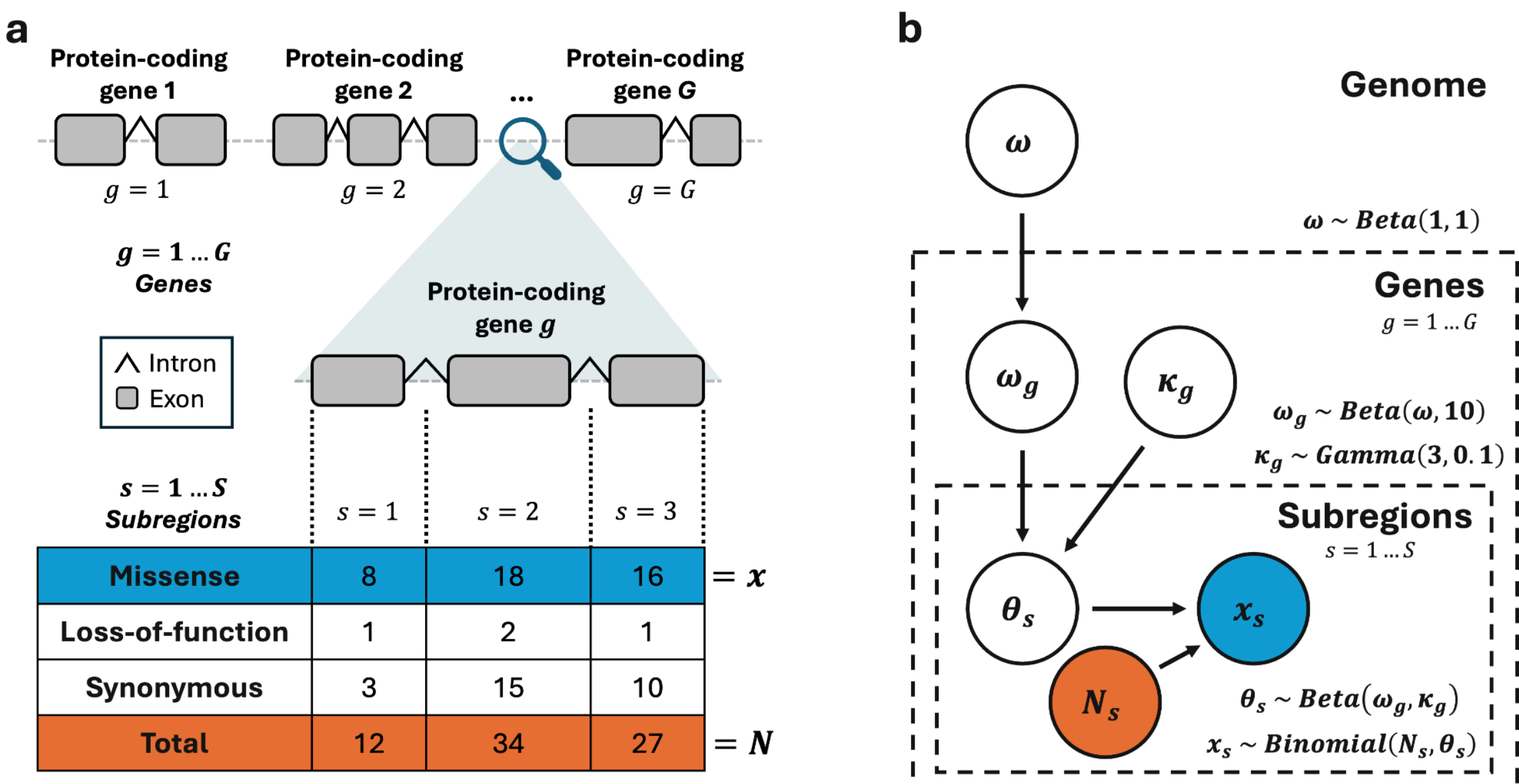

Supplementary Figure 1: PRIME Framework and Model for Exon-Based Subregions.
a, Workflow to obtain regional variant counts for exons. Protein-coding genes are divided into exons based on Ensembl annotations, and gnomAD exome and genome variants are counted in each region after consequence annotation. **b**, Graphical model of PRIME, fit to obtained variant counts from previous step. Colored nodes denote observed data and unfilled nodes denote latent parameters. PRIME jointly estimates θ, the probability of a variant in the population within an exon being missense. Hyperpriors match those used for the domain-based model in Fig. 1b.

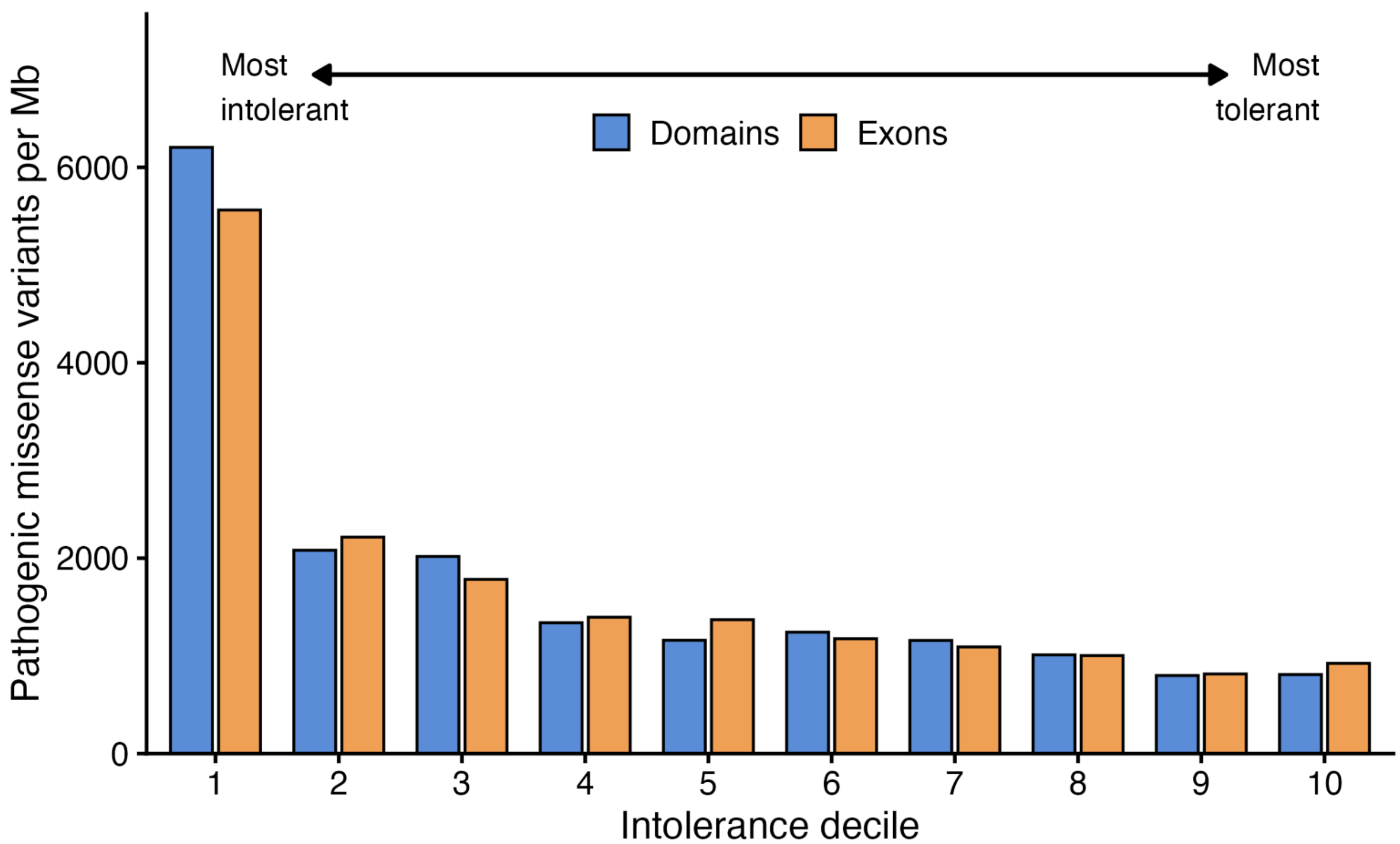

Supplementary Figure 2: Pathogenic Variants per Megabase by Intolerance Deciles.
ClinVar pathogenic missense variant counts per megabase for equally sized deciles of intolerance. Intolerant regions demonstrate a higher rate of pathogenic variants relative to more tolerant regions across the genome for both domain and exon-based regions.

Gene Set	Weakest Link (Domains)	Weakest Link (Exons)	MCR O/E Minimum	Missense Z	MOEUF	RVIS	Omega HDI Upper Bound
De Novo	0.749 [0.733, 0.764]	**0.759 [0.744, 0.774]**	0.747 [0.733, 0.762]	0.750 [0.735, 0.766]	0.740 [0.726, 0.756]	0.743 [0.727, 0.758]	0.725 [0.709, 0.740]
Gain-of-Function	**0.737 [0.713, 0.759]**	0.725 [0.700, 0.749]	0.725 [0.700, 0.750]	0.726 [0.701, 0.750]	0.714 [0.691, 0.737]	0.727 [0.702, 0.753]	0.730 [0.704, 0.754]
Haploinsufficient	0.727 [0.694, 0.758]	0.729 [0.696, 0.761]	0.704 [0.669, 0.737]	0.671 [0.633, 0.708]	0.661 [0.626, 0.696]	**0.738 [0.702, 0.771]**	0.702 [0.666, 0.735]
Dominant-Negative	0.711 [0.690, 0.731]	0.702 [0.681, 0.723]	0.708 [0.686, 0.728]	**0.714 [0.691, 0.733]**	0.704 [0.684, 0.724]	0.697 [0.674, 0.719]	0.695 [0.674, 0.716]
Autosomal Dominant	**0.710 [0.697, 0.724]**	0.703 [0.688, 0.716]	0.704 [0.690, 0.717]	0.697 [0.682, 0.712]	0.687 [0.673, 0.702]	0.707 [0.692, 0.722]	0.702 [0.688, 0.716]
All OMIM	0.586 [0.576, 0.595]	**0.618 [0.608, 0.627]**	0.567 [0.557, 0.577]	0.575 [0.565, 0.585]	0.570 [0.561, 0.580]	0.567 [0.557, 0.578]	0.596 [0.586, 0.606]
Autosomal Recessive	0.494 [0.483, 0.505]	**0.540 [0.528, 0.550]**	0.464 [0.452, 0.475]	0.480 [0.469, 0.491]	0.475 [0.465, 0.485]	0.471 [0.458, 0.483]	0.523 [0.512, 0.535]

Supplementary Table 1: AUROCs of Intolerance Metrics Discriminating OMIM Disease Gene Sets.
AUROCs of intolerance metrics with 95% confidence intervals in brackets. Confidence intervals were estimated using 2,000 bootstrap resamples of each dataset. Top performing method based on AUROC for a given gene set is bolded.

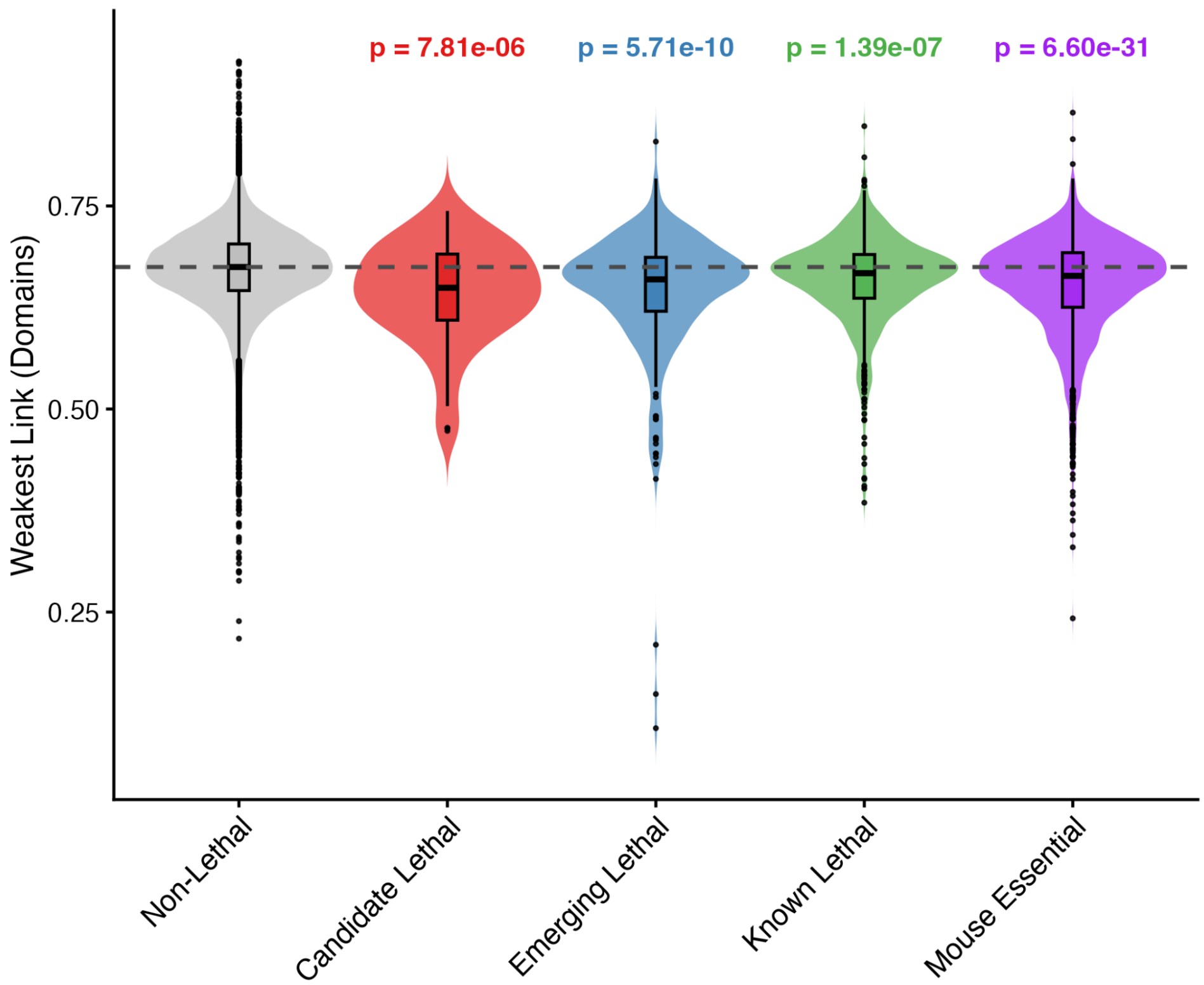

Supplementary Figure 3: Lethal/Essential Genes Domain-Based Weakest Link Intolerance. Distribution of the domain-based weakest link statistic for lethal genes with varying levels of evidence and genes essential for development in mice. Above p-values are from one-sided Mann-Whitney tests comparing the distributional differences between the lethal/essential gene sets and the complement of those sets combined (non-lethal genes). Dashed line represents the median weakest link value among non-lethal group.

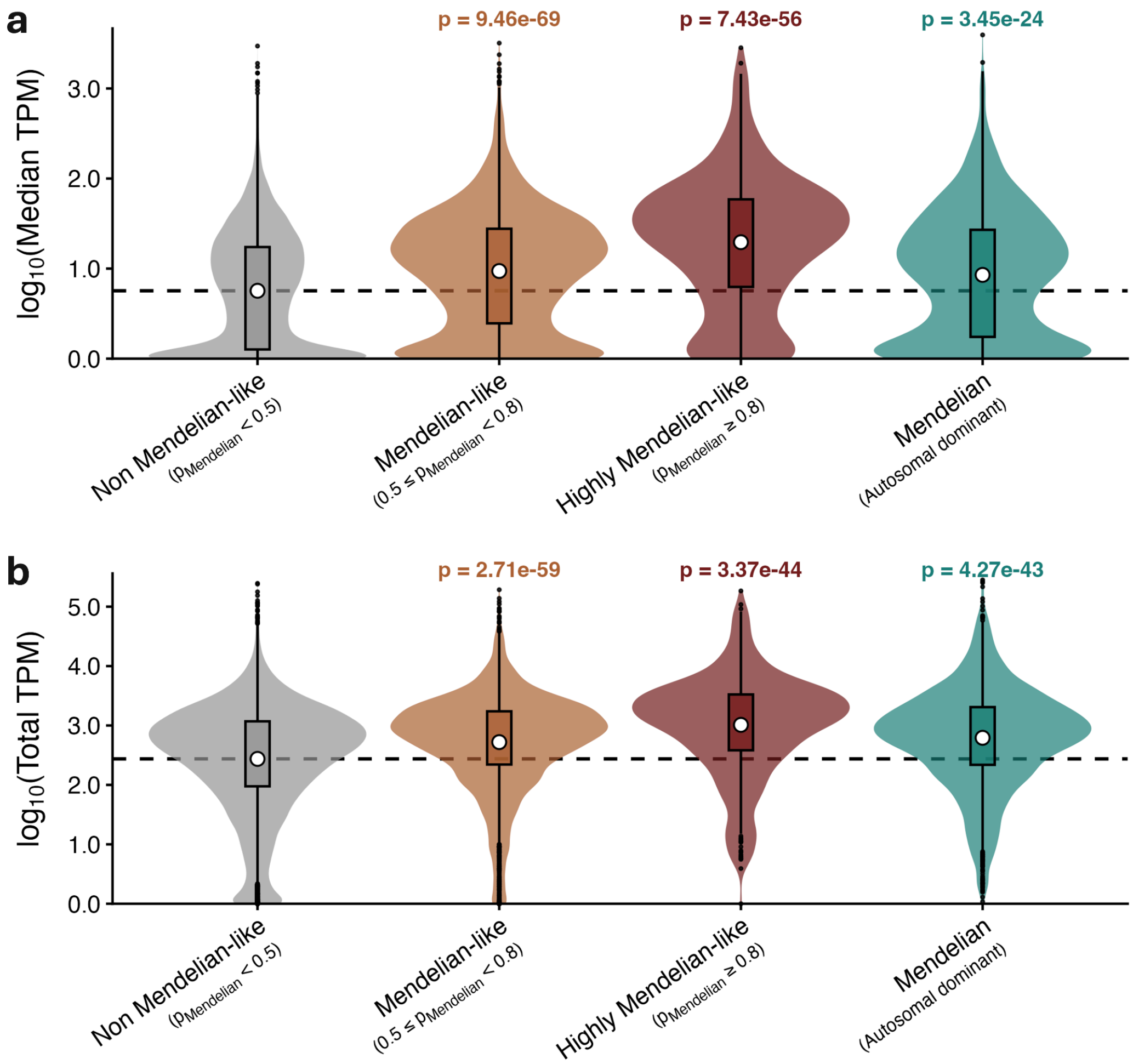

Supplementary Figure 4: Distribution of Domain-based Mendelian-like and Non Mendelian-like Gene Expression.

a, Mendelian-like genes and known Mendelian disease genes show higher median expression across tissue types compared to non-Mendelian genes. **b,** Mendelian-like genes and known Mendelian disease genes show higher total expression across tissue types compared to non-Mendelian genes. Dashed lines represent the mean values of the non Mendelian-like set.

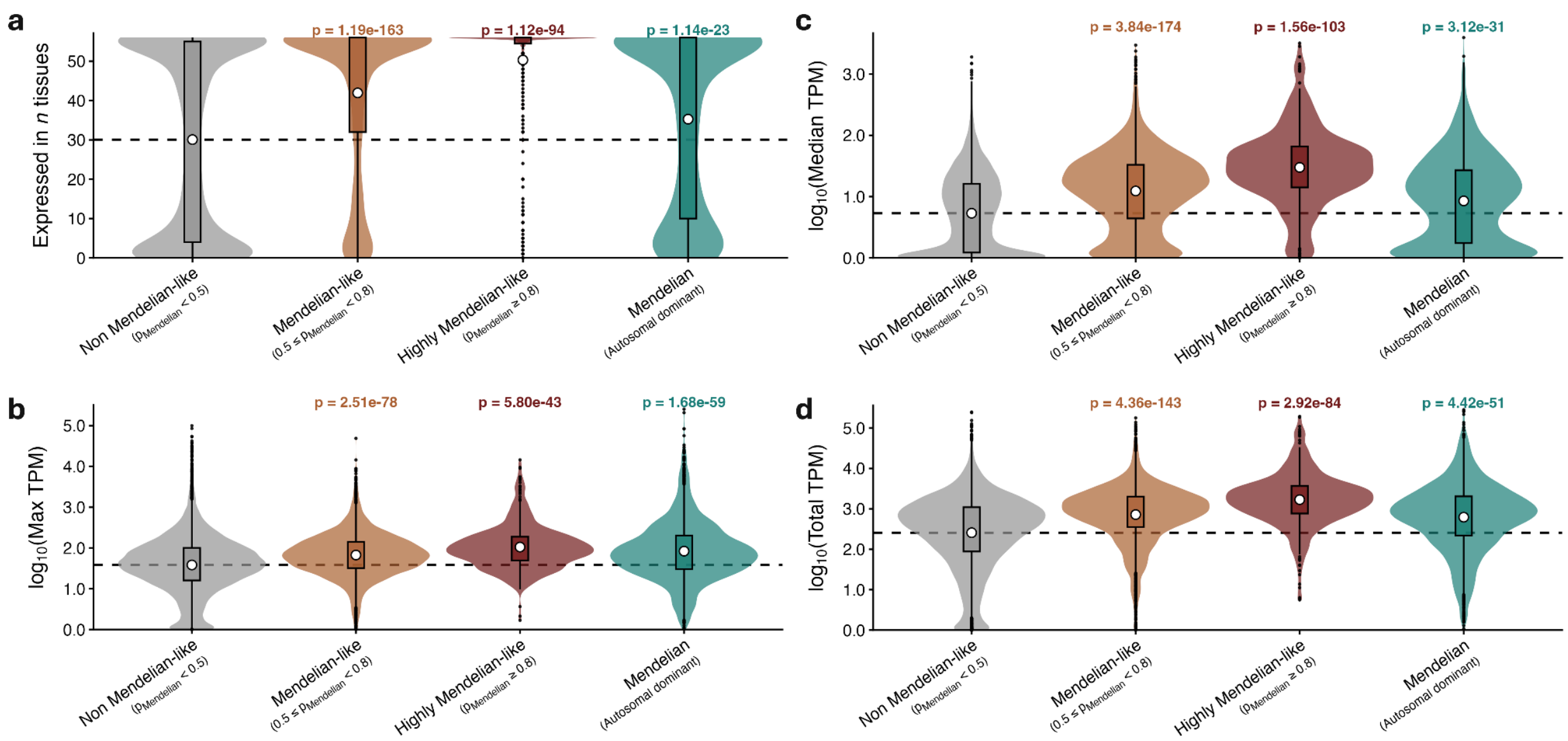

Supplementary Figure 5: Distribution of Exon-based Mendelian-like and Non Mendelian-like Gene Expression.

a, Number of tissues expressed, **b,** maximum tissue-specific expression, **c,** median expression across tissues, and **d,** total expression across tissues for non-Mendelian, Mendelian-like, and known Mendelian disease genes. Mendelian-like genes and known Mendelian disease genes exhibit higher expression across all four measures. Dashed lines represent the mean values of the non-Mendelian like gene set.

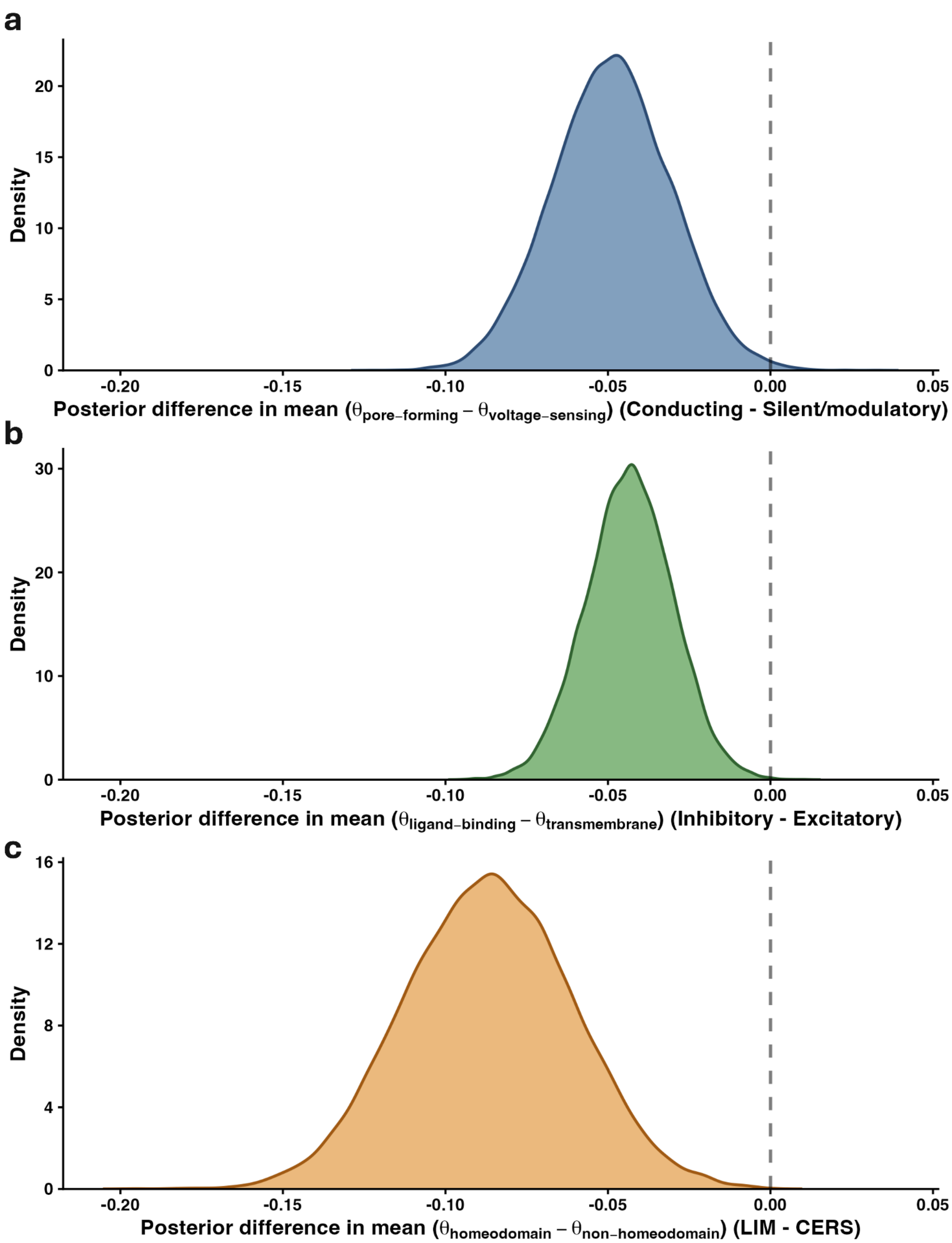

Supplementary Figure 6: Posterior Distributions of the Difference in Means Between Gene Family Subgroups.

a, Conducting Kv channels display a stronger difference in intolerance between the pore-forming and voltage-sensing domains relative to silent/modulatory Kv channels, with 99.63% of computed posterior samples below zero. **b,** Inhibitory cys-loop receptors display more intolerance on average in their ligand-binding domain than their transmembrane domain compared to excitatory receptors, with 99.94% of computed posterior samples below zero. **c,** LIM homeoboxes display more localized intolerance on average in their DNA-binding homeodomains compared to CERS homeoboxes, with 99.99% of computed posterior samples below zero.

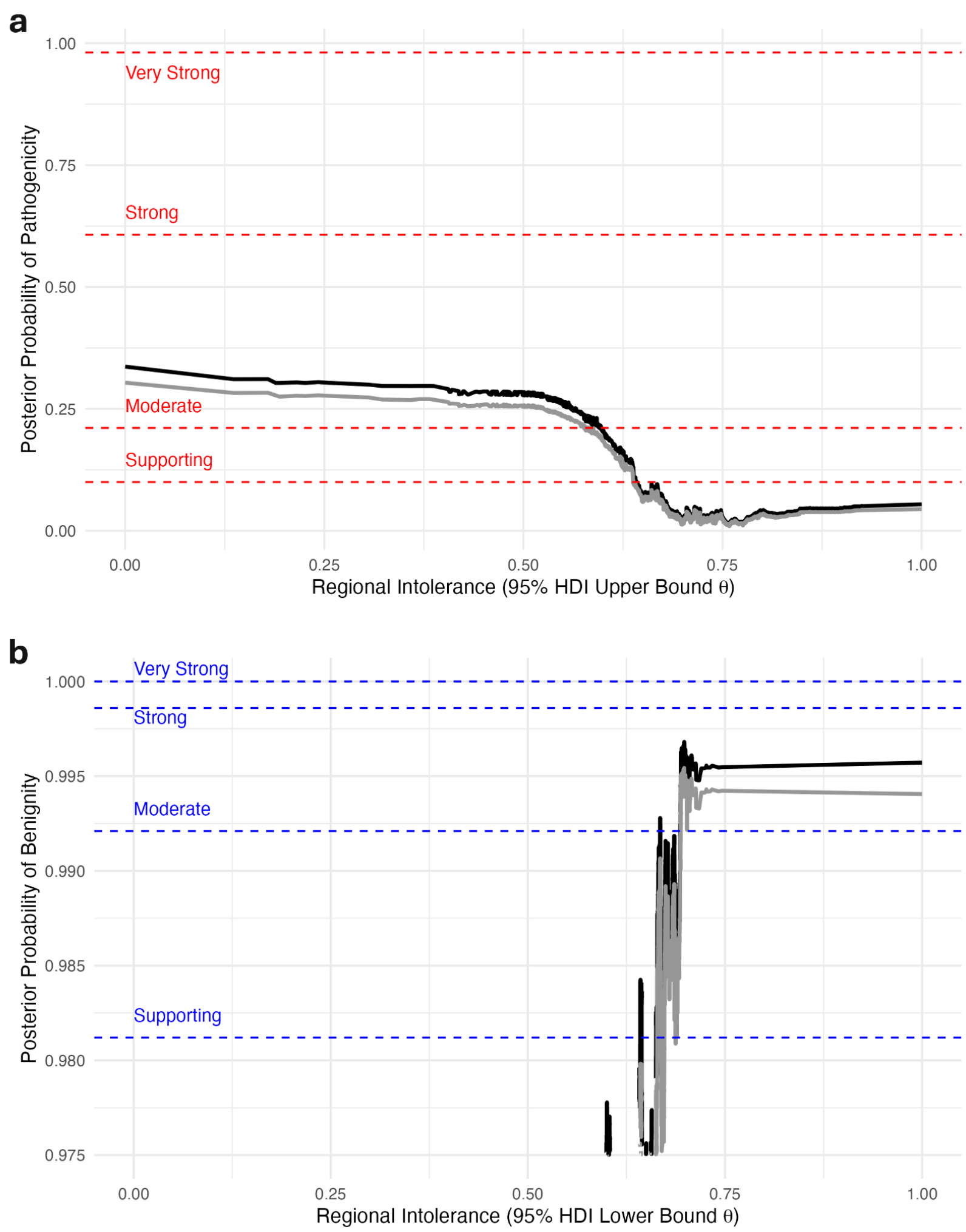

Supplementary Figure 7: Calibration of PRIME Estimates for Pathogenicity and Benignity.
a, Local posterior probabilities of pathogenicity given the upper bound of the 95% HDI of θ. This estimate is conservative in the direction of intolerance. **b**, Local posterior probabilities of benignity given the lower bound of the 95% HDI of θ. This estimate is conservative in the direction of tolerance.

Evidence	PRIME summary statistic	Supporting	Moderate
Pathogenic	$\theta_{HDIupper}$	(0.605139, 0.645456]	≤ 0.605139
Benign	$\theta_{HDIlower}$	[0.670441, 0.693056)	≥ 0.693056

Supplementary Table 2: Calibrated PRIME Thresholds of Variant Pathogenicity and Benignity. Supporting and moderate evidence thresholds of pathogenicity and benignity determined by 10,000 bootstrap iterations. “(“ and “)” denote exclusion of the end value and “[“ and “]” denote inclusion.

Predictor	Strength	Predictor only	+ θ supporting	+ θ moderate
BayesDel	Supporting	3.23	7.14	20.95
BayesDel	Moderate	8.48	17.14	49.77
BayesDel	Strong	51.73	107.1	143.19
CADD	Supporting	3.39	7.73	31.93
CADD	Moderate	6.03	18.79	48.12
EA	Supporting	3.87	13.13	58.75
EA	Moderate	12.02	195.83	99.64
FATHMM	Supporting	3.64	7.18	17.88
FATHMM	Moderate	14.5	24.77	36.38
MPC	Supporting	2.79	3	4.63
MPC	Moderate	9.59	8.77	22.21
MutPred2	Supporting	3.92	4.16	11.18
MutPred2	Moderate	9.58	16.73	28.26
MutPred2	Strong	44.88	59.38	62.79
PhyloP	Supporting	4.11	7.27	27.8
PhyloP	Moderate	5.69	8.87	15.49
PolyPhen2	Supporting	2.85	10.22	34.66
PolyPhen2	Moderate	9.76	28.18	174.69
PrimateAI	Supporting	3.4	4.11	14.9
PrimateAI	Moderate	8.19	12.57	46.23
REVEL	Supporting	3.77	5.19	20.38
REVEL	Moderate	9.72	21.15	45.91
REVEL	Strong	52.81	102.14	139.29
SIFT	Supporting	3.31	7.48	54.2
SIFT	Moderate	6.04	23.23	30.51
VEST4	Supporting	3.31	5.75	20.69
VEST4	Moderate	9.56	16.93	74.67
VEST4	Strong	35.75	100.63	103.02

Supplementary Table 3: Pathogenicity Likelihood Ratios in ClinVar 2020 Dataset.
Interval-based positive likelihood ratios for the pathogenic predictor intervals alone, predictor intervals further stratified by $\theta_{HDIupper}$ supporting evidence, and predictor intervals further stratified by $\theta_{HDIupper}$ moderate evidence. Higher values indicate higher probability of pathogenicity.

Predictor	Strength	Predictor only	+ θ supporting	+ θ moderate
BayesDel	Supporting	0.23	0.23	0.05
BayesDel	Moderate	0.06	0.06	0.03
CADD	Supporting	0.22	0.14	0.05
CADD	Moderate	0.06	0.05	0.02
CADD	Strong	0	0	0
EA	Supporting	0.17	0.18	0.06
EA	Moderate	0.06	0.05	0.03
FATHMM	Supporting	0.15	0	0
FATHMM	Moderate	0.05	0	0.07
GERP	Supporting	0.16	0.12	0.02
GERP	Moderate	0.12	0.06	0
MutPred2	Supporting	0.21	0.27	0.14
MutPred2	Moderate	0.07	0.09	0.03
MutPred2	Strong	0	0	0
PhyloP	Supporting	0.21	0.17	0.03
PhyloP	Moderate	0.09	0.04	0.02
PolyPhen2	Supporting	0.2	0.2	0.02
PolyPhen2	Moderate	0.1	0.06	0.01
PrimateAI	Supporting	0.21	0.34	0.06
PrimateAI	Moderate	0.07	0.11	0.03
REVEL	Supporting	0.24	0.3	0.06
REVEL	Moderate	0.06	0.06	0.03
REVEL	Strong	0	0	0
REVEL	Very strong	0	0	0
SIFT	Supporting	0.2	0.26	0.07
SIFT	Moderate	0.08	0.03	0.04
VEST4	Supporting	0.16	0.22	0.08
VEST4	Moderate	0.06	0.1	0.02

Supplementary Table 4: Benignity Likelihood Ratios in ClinVar 2020 Dataset.
Interval-based positive likelihood ratios for the benign predictor intervals alone, predictor intervals further stratified by $\theta_{HDIupper}$ supporting evidence, and predictor intervals further stratified by $\theta_{HDIupper}$ moderate evidence. Lower values indicate lower probability of pathogenicity, and thus higher probability of benignity.

Predictor	Strength	Predictor only coverage	+ θ supporting coverage	+ θ moderate coverage	Predictor only counts	+ θ supporting counts	+ θ moderate counts	Total counts
BayesDel	Supporting	7.9%	0.7%	1.1%	381 path / 275 benign	46 path / 15 benign	81 path / 9 benign	2498 path / 5816 benign
BayesDel	Moderate	11.6%	1.1%	2.2%	758 path / 208 benign	81 path / 11 benign	171 path / 8 benign	2498 path / 5816 benign
BayesDel	Strong	8.9%	1.1%	1.5%	711 path / 32 benign	92 path / 2 benign	123 path / 2 benign	2498 path / 5816 benign
CADD	Supporting	17.1%	1.5%	2.5%	842 path / 579 benign	93 path / 28 benign	192 path / 14 benign	2498 path / 5816 benign
CADD	Moderate	16.1%	1.5%	2.3%	966 path / 373 benign	113 path / 14 benign	186 path / 9 benign	2498 path / 5816 benign
EA	Supporting	9.8%	0.9%	1.4%	465 path / 277 benign	57 path / 10 benign	102 path / 4 benign	2284 path / 5262 benign
EA	Moderate	14.1%	1.1%	2.3%	892 path / 171 benign	85 path / 1 benign	173 path / 4 benign	2284 path / 5262 benign
FATHMM	Supporting	5.6%	0.6%	1.2%	249 path / 159 benign	34 path / 11 benign	77 path / 10 benign	2186 path / 5076 benign
FATHMM	Moderate	7.4%	1.0%	0.7%	462 path / 74 benign	64 path / 6 benign	47 path / 3 benign	2186 path / 5076 benign
MPC	Supporting	7.7%	1.0%	0.9%	266 path / 209 benign	37 path / 27 benign	38 path / 18 benign	1934 path / 4240 benign
MPC	Moderate	14.0%	2.6%	5.6%	704 path / 161 benign	128 path / 32 benign	314 path / 31 benign	1934 path / 4240 benign
MutPred2	Supporting	7.0%	0.7%	0.8%	366 path / 216 benign	36 path / 20 benign	58 path / 12 benign	2495 path / 5772 benign
MutPred2	Moderate	11.8%	1.3%	2.2%	787 path / 190 benign	94 path / 13 benign	171 path / 14 benign	2495 path / 5772 benign
MutPred2	Strong	8.6%	1.0%	2.4%	679 path / 35 benign	77 path / 3 benign	190 path / 7 benign	2495 path / 5772 benign
PhyloP	Supporting	23.8%	2.6%	4.1%	1237 path / 706 benign	158 path / 51 benign	308 path / 26 benign	2443 path / 5734 benign
PhyloP	Moderate	4.1%	0.5%	0.9%	235 path / 97 benign	34 path / 9 benign	66 path / 10 benign	2443 path / 5734 benign
PolyPhen2	Supporting	14.2%	1.2%	1.8%	564 path / 446 benign	68 path / 15 benign	123 path / 8 benign	2181 path / 4916 benign
PolyPhen2	Moderate	13.1%	1.1%	2.2%	758 path / 175 benign	75 path / 6 benign	155 path / 2 benign	2181 path / 4916 benign
PrimateAI	Supporting	12.8%	1.5%	2.0%	594 path / 408 benign	74 path / 42 benign	134 path / 21 benign	2355 path / 5499 benign
PrimateAI	Moderate	12.5%	2.1%	4.0%	765 path / 218 benign	140 path / 26 benign	297 path / 15 benign	2355 path / 5499 benign
REVEL	Supporting	6.8%	0.7%	1.1%	343 path / 211 benign	38 path / 17 benign	79 path / 9 benign	2442 path / 5669 benign
REVEL	Moderate	11.6%	1.1%	2.3%	762 path / 182 benign	82 path / 9 benign	178 path / 9 benign	2442 path / 5669 benign
REVEL	Strong	8.2%	1.1%	1.5%	637 path / 28 benign	88 path / 2 benign	120 path / 2 benign	2442 path / 5669 benign
SIFT	Supporting	8.0%	0.5%	1.0%	333 path / 234 benign	29 path / 9 benign	70 path / 3 benign	2128 path / 4943 benign
SIFT	Moderate	20.5%	1.9%	3.0%	1048 path / 403 benign	120 path / 12 benign	197 path / 15 benign	2128 path / 4943 benign
VEST4	Supporting	9.4%	0.9%	1.4%	426 path / 308 benign	48 path / 20 benign	95 path / 11 benign	2291 path / 5489 benign
VEST4	Moderate	14.6%	1.6%	2.5%	906 path / 227 benign	106 path / 15 benign	187 path / 6 benign	2291 path / 5489 benign
VEST4	Strong	5.1%	0.6%	0.6%	373 path / 25 benign	42 path / 1 benign	43 path / 1 benign	2291 path / 5489 benign

Supplementary Table 5: Pathogenicity Threshold Coverage of ClinVar 2020 Variants. Coverage of total variants in the dataset for the pathogenic predictor intervals alone, predictor intervals further stratified by $\theta_{HDIupper}$ supporting evidence, and predictor intervals further stratified by $\theta_{HDIupper}$ moderate evidence.

Predictor	Strength	Predictor only coverage	+ θ supporting coverage	+ θ moderate coverage	Predictor only counts	+ θ supporting counts	+ θ moderate counts	Total counts
BayesDel	Supporting	17.8%	1.8%	1.1%	131 path / 1351 benign	13 path / 134 benign	2 path / 88 benign	2498 path / 5816 benign
BayesDel	Moderate	32.0%	4.3%	3.8%	63 path / 2594 benign	9 path / 349 benign	4 path / 312 benign	2498 path / 5816 benign
CADD	Supporting	18.6%	1.7%	1.2%	131 path / 1418 benign	8 path / 134 benign	2 path / 101 benign	2498 path / 5816 benign
CADD	Moderate	23.9%	2.9%	2.8%	47 path / 1938 benign	5 path / 237 benign	2 path / 232 benign	2498 path / 5816 benign
CADD	Strong	2.6%	0.4%	0.5%	0 path / 217 benign	0 path / 34 benign	0 path / 43 benign	2498 path / 5816 benign
EA	Supporting	25.5%	2.5%	2.1%	130 path / 1791 benign	14 path / 177 benign	4 path / 151 benign	2284 path / 5262 benign
EA	Moderate	13.6%	1.8%	1.2%	28 path / 999 benign	3 path / 130 benign	1 path / 87 benign	2284 path / 5262 benign
FATHMM	Supporting	2.3%	0.4%	0.6%	10 path / 160 benign	0 path / 27 benign	0 path / 42 benign	2186 path / 5076 benign
FATHMM	Moderate	0.7%	0.1%	0.5%	1 path / 49 benign	0 path / 4 benign	1 path / 35 benign	2186 path / 5076 benign
GERP	Supporting	22.3%	2.3%	2.6%	119 path / 1702 benign	9 path / 176 benign	2 path / 212 benign	2442 path / 5730 benign
GERP	Moderate	3.8%	0.5%	0.5%	15 path / 298 benign	1 path / 42 benign	0 path / 40 benign	2442 path / 5730 benign
MutPred2	Supporting	17.4%	1.8%	1.1%	120 path / 1316 benign	16 path / 135 benign	5 path / 84 benign	2495 path / 5772 benign
MutPred2	Moderate	34.1%	4.3%	3.9%	86 path / 2731 benign	14 path / 343 benign	4 path / 321 benign	2495 path / 5772 benign
MutPred2	Strong	0.1%	0.0%	0.0%	0 path / 8 benign	0 path / 3 benign	0 path / 0 benign	2495 path / 5772 benign
PhyloP	Supporting	22.9%	2.7%	2.2%	156 path / 1717 benign	15 path / 203 benign	2 path / 179 benign	2443 path / 5734 benign
PhyloP	Moderate	11.1%	1.4%	1.5%	35 path / 874 benign	2 path / 110 benign	1 path / 125 benign	2443 path / 5734 benign
PolyPhen2	Supporting	15.4%	1.6%	1.5%	90 path / 1004 benign	9 path / 103 benign	1 path / 102 benign	2181 path / 4916 benign
PolyPhen2	Moderate	22.9%	2.6%	2.3%	66 path / 1558 benign	5 path / 176 benign	1 path / 162 benign	2181 path / 4916 benign
PrimateAI	Supporting	14.5%	1.8%	1.1%	94 path / 1047 benign	18 path / 122 benign	2 path / 84 benign	2355 path / 5499 benign
PrimateAI	Moderate	20.9%	3.3%	3.6%	47 path / 1594 benign	12 path / 247 benign	4 path / 280 benign	2355 path / 5499 benign
REVEL	Supporting	13.8%	1.3%	0.9%	105 path / 1011 benign	12 path / 94 benign	2 path / 72 benign	2442 path / 5669 benign
REVEL	Moderate	32.8%	4.1%	3.8%	70 path / 2590 benign	9 path / 325 benign	4 path / 307 benign	2442 path / 5669 benign
REVEL	Strong	1.6%	0.3%	0.3%	0 path / 132 benign	0 path / 25 benign	0 path / 25 benign	2442 path / 5669 benign
REVEL	Very strong	0.1%	0.0%	0.1%	0 path / 12 benign	0 path / 4 benign	0 path / 5 benign	2442 path / 5669 benign
SIFT	Supporting	20.3%	2.1%	1.5%	113 path / 1322 benign	15 path / 134 benign	3 path / 102 benign	2128 path / 4943 benign
SIFT	Moderate	18.6%	2.1%	1.6%	43 path / 1272 benign	2 path / 145 benign	2 path / 112 benign	2128 path / 4943 benign
VEST4	Supporting	12.6%	1.2%	0.8%	63 path / 918 benign	8 path / 89 benign	2 path / 58 benign	2291 path / 5489 benign
VEST4	Moderate	36.0%	4.4%	3.9%	63 path / 2739 benign	14 path / 332 benign	3 path / 303 benign	2291 path / 5489 benign

Supplementary Table 6: Benignity Threshold Coverage of ClinVar 2020 Variants.
Coverage of total variants in the dataset for the benign predictor intervals alone, predictor intervals further stratified by $\theta_{HDIlower}$ supporting evidence, and predictor intervals further stratified by $\theta_{HDIlower}$ moderate evidence.

Gene Set	Autosomal Dominant	Autosomal Recessive	Dominant-Negative	Haploinsufficient	Gain-of-Function	De Novo
**Autosomal Dominant**	1606 (100.0%)	459 (28.6%)	547 (34.1%)	225 (14.0%)	406 (25.3%)	989 (61.6%)
**Autosomal Recessive**	459 (16.2%)	2830 (100.0%)	283 (10.0%)	79 (2.8%)	166 (5.9%)	406 (14.3%)
**Dominant-Negative**	547 (80.9%)	283 (41.9%)	676 (100.0%)	100 (14.8%)	183 (27.1%)	429 (63.5%)
**Haploinsufficient**	225 (81.2%)	79 (28.5%)	100 (36.1%)	277 (100.0%)	63 (22.7%)	192 (69.3%)
**Gain-of-Function**	406 (83.5%)	166 (34.2%)	183 (37.7%)	63 (13.0%)	486 (100.0%)	321 (66.0%)
**De Novo**	989 (75.8%)	406 (31.1%)	429 (32.9%)	192 (14.7%)	321 (24.6%)	1305 (100.0%)

Supplementary Table 7: OMIM Gene Set Overlap.

Overlap of gene counts within derived OMIM disease gene sets. Percentages are computed for the gene set in the corresponding row. Genes may have both dominant and recessive phenotypes in OMIM, explaining the overlap of gene sets with dominant effects with the autosomal recessive group.
